# Karyotype Diversification and Chromosome Rearrangements in Squamate Reptiles

**DOI:** 10.3390/genes15030371

**Published:** 2024-03-18

**Authors:** Marcello Mezzasalma, Rachele Macirella, Gaetano Odierna, Elvira Brunelli

**Affiliations:** 1 Department of Biology, Ecology and Earth Science, University of Calabria, Via P. Bucci 4/B, 87036 Rende, Italy; rachele.macirella@unical.it (R.M.); elvira.brunelli@unical.it (E.B.); 2 Independent Researcher, Via Michelangelo 123, 81031 Aversa, Italy; gaetanodierna@gmail.com

**Keywords:** cytogenetics, evolution, macromutations, phylogeny, reptiles

## Abstract

Karyotype diversification represents an important, yet poorly understood, driver of evolution. Squamate reptiles are characterized by a high taxonomic diversity which is reflected at the karyotype level in terms of general structure, chromosome number and morphology, and insurgence of differentiated simple or multiple-sex-chromosome systems with either male or female heterogamety. The potential of squamate reptiles as unique model organisms in evolutionary cytogenetics has been recognised in recent years in several studies, which have provided novel insights into the chromosome evolutionary dynamics of different taxonomic groups. Here, we review and summarize the resulting complex, but promising, general picture from a systematic perspective, mapping some of the main squamate karyological characteristics onto their phylogenetic relationships. We highlight how all the major categories of balanced chromosome rearrangements contributed to the karyotype evolution in different taxonomic groups. We show that distinct karyotype evolutionary trends may occur, and coexist, with different frequencies in different clades. Finally, in light of the known squamate chromosome diversity and recent research advances, we discuss traditional and novel hypotheses on karyotype evolution and propose a scenario of circular karyotype evolution.

## 1. Introduction

Chromosome evolution is a major source of biodiversity. Changes in the karyotype structure occur via chromosome rearrangements (macromutations) which directly affect chromosome number and morphology. At a low taxonomic level, the role of chromosome changes in building genetic barriers and promoting speciation has been much debated in recent decades, but a growing amount of evidence highlights that all the main categories of chromosome rearrangements may have an active role in preventing gene flow and promoting lineage diversification (e.g., [1,2,3,4,5]). However, in contrast to the well-established connection between chromosome changes and taxonomic and phylogenetic diversification, karyotype evolution still remains largely understudied and poorly understood.

Squamate reptiles represent a highly successful and diverse monophyletic group of vertebrates including more than 11.000 currently described species, which are classified into more than 42 lizard and 32 snake families [6]. Lizards and snakes are characterized by a continuously updated taxonomy and complex phylogenetic relationships and by a high number of newly described species in the last few years (>500 between 2020 and 2023) [6,7]. Given their high biological and taxonomic diversity, squamates represent particularly promising model organisms in different fields of study such as evolutionary biology and genetics, evo-devo, historical biogeography, and conservation and invasion biology (e.g., [8,9,10,11]).

From a cytogenetic point of view, the high taxonomic and biological diversity of squamate reptiles is reflected at the chromosome level. Squamate reptiles show a remarkable diversity in their karyotype structure, including chromosome number and morphology, presence or absence of different chromosome classes (micro- and macrochromosomes) and the number and localization of specific chromosome markers (see, e.g., [12,13]). Chromosome sex determination systems are also highly variable in squamates. Different families include simple and/or multiple-sex-chromosome systems with either male (XX/XY) or female heterogamety (ZZ/ZZ), encompassing every hypothesized diversification stage of heterogametic sex chromosomes, from homomorphic and pseudo-autosomal to heteromorphic and completely heterochromatic chromosomes [14,15].

These characteristics identify squamate reptiles as a particularly suited vertebrate group to better understand chromosome evolutionary dynamics at both micro- and macroevolutionary level. This potential has been widely recognized in recent years by a growing number of studies, which have provided new insights into several taxonomic groups (e.g., [16,17,18,19]). In this contribution we summarize the resulting general picture which represents a challenging, but promising, frontier in evolutionary cytogenetics.

In the first part of this review we describe the general karyotype structure of squamate reptiles from a systematic perspective, mapping some of their major karyological characteristics onto their phylogenetic relationships (see, e.g., [6,7]). We discuss their general and lineage-specific karyological diversity, highlighting the occurrence and distribution of plesiomorphic and apomorphic chromosome features. Then, we discuss the impact and the evolutionary role of the main categories of balanced chromosome rearrangements (chromosome fusions, fissions and inversions), providing some explanatory examples at different taxonomic levels. In the last part of this contribution, we focus on the main observed evolutionary trends and pathways of karyotype diversification in squamates taking into consideration previously advanced hypotheses and proposing a virtual scenario of circular karyotype evolution. We highlight that a better understanding of the chromosome diversity and evolution of squamate reptiles would represent a critical advancement in our interpretation of the biological processes of karyotype and genome diversification in vertebrates and, more in general, of life itself.

## 2. Karyotype Structure and Variability in Squamates

**Figure 1:**
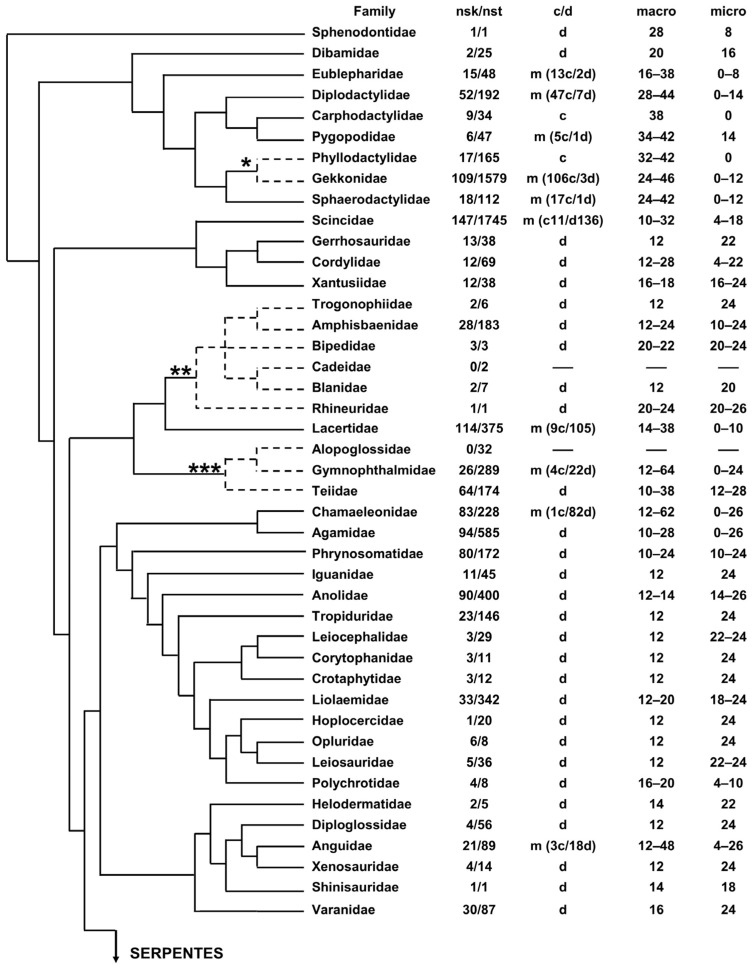
Phylogenetic relationships of lizard families + *Sphenodon* superimposed with karyotype data. nsk = number of species with a described karyotype; nst = number of described species; c = continuous karyotype; d = discontinuous karyotype; m = mix of continuous and discontinuous karyotypes; micro = number of microchromosome pairs; macro = number of macrochromosome pairs (chromosome data were gathered from [6] and therein references). Phylogenetic relationships redrawn from [29]. Dashed lines represent phylogenetic relationships by * [30], ** [31] and *** [32].

The squamate genome presents some peculiar features which are widely shared among different families. For example, in contrast to what is commonly observed in mammals and amphibians, lizards and snakes are characterized by a relatively small and homogeneous genome size with a low content of heterochromatin [12,20]. In fact, the karyotypes of squamate reptiles do not usually exhibit a banding pattern similar to that observed in other vertebrates, and most heterochromatin is localized on telomeric or centromeric regions, or, when present, on differentiated heterogametic sex chromosomes (Y/W) and B chromosomes (e.g., [12,21,22]). Squamates nevertheless encompass a high variability in chromosome number with diploid karyotypes ranging from 2n = 16 (in the gekkonid *Gonatodes taniae*) to 2n = 62 (in the chameleon *Rieppeleon brevicaudatus* and the microteid *Notobrachia ablephara* [23,24,25]). Chromosome morphology is also highly variable in squamates, with different clades showing only uniarmed (acrocentric), biarmed (meta-, submeta- and subtelocentric) elements or a mix of a variable number of uni- and biarmed elements [12,15]. It should be noted that this high variability is included in two main different types of karyotype structure, defined as discontinuous (or bimodal) and continuous (or unimodal) karyotypes. Discontinuous karyotypes are those with chromosomes that are clearly distinguishable in two dimensional classes (macro- and microchromosomes), while continuous karyotypes are composed of chromosomes gradually decreasing in size, without a clear difference between macro- and microchromosomes [26]. Discontinuous karyotypes are thought to represent an ancestral condition in vertebrates and are mostly found in squamates, birds, turtles, and some basal fish and amphibian clades, while continuous karyotypes occur in most amphibian, teleost, mammal and some reptile taxa (see, e.g., [27,28]). Among lizard families (see [29,30,31,32,33] for phylogenetic hypotheses), 28 show only discontinuous karyotypes, 9 are characterized by a mix of continuous and discontinuous karyotypes, while just Carphodactylidae and Phyllodactylidae display only karyotypes without microchromosomes (Figure 1). However, the presence of continuous karyotypes is particularly common in Gekkota, where they are displayed by the majority of species, as well as in families with a mixed karyotype structure (Eublepharidae, Diplodactylidae, Pygopodidae, Gekkonidae and Sphaerodactylidae) (Figure 1).

Most lizard families (n = 29) also show a variable number of macro- and/or microchromosomes while another 16 families display a fixed chromosome formula (Figure 1). However, those showing a constant chromosome complement often have a low number of described species and/or karyotypes (Figure 1). One exception is represented by Varanidae, where all the species with a known karyotype show a highly conserved chromosome formula of 2n = 40 with 8 macro- and 12 microchromosome pairs [16].

The degree of chromosome diversity in squamates may also be highly variable among genera of the same family. For example, the family Chamaeleonidae includes the genera *Brookesia* and *Palleon* with a fixed chromosome formula (14 karyotyped species, all with 2n = 36) as well as *Furcifer* which shows a high karyotype variability (21 karyotyped species with 2n = 22–34) (see, e.g., [19,25]).

**Figure 2:**
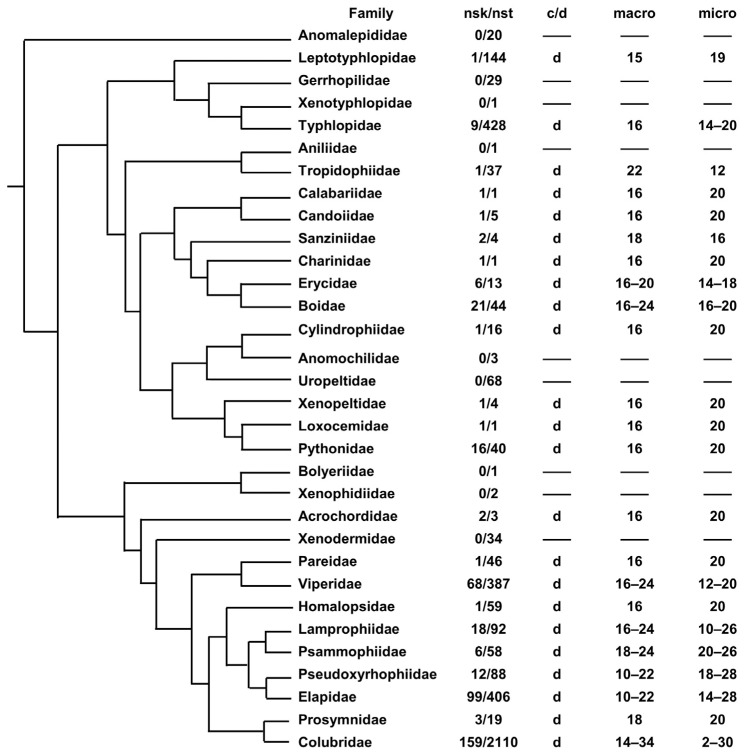
Phylogenetic relationships of snake families superimposed with karyotype data. nsk = number of species with a described karyotype; nst = number of described species; c = continuous karyotype; d = discontinuous karyotype; m = mix of continuous and discontinuous karyotypes; micro = number of microchromosome pairs; macro = number of macrochromosome pairs (chromosome data were gathered from [6] and therein references). Phylogenetic relationships redrawn from [33,34].

In snake phylogeny (see [33,34] for phylogenetic hypotheses), chromosome variability appears to be, overall, lower than in lizards (Figure 2). They show only discontinuous karyotypes; in fact, several species which have been occasionally reported as having only macrochromosomes (e.g., *Erythrolamprus, Helicops* and *Hydrodynastes*) show at least one microchromosome pair [35,36,37]. A total of 8 families have a variable chromosome number and 14 families display a fixed karyotype formula (2n = 36, with 8 macro- and 10 microchromosome pairs in 9 families) (Figure 2). It should be noted that, even in this case, a fixed chromosome complement is often displayed by clades that include a low number of described species and/or a low number of species with a known karyotype (Figure 2).

Given the diversity of the chromosome complement of squamates it is difficult to reliably describe the ancestral karyotype of all their main evolutionary branches. However, various insights from traditional and molecular cytogenetics indicate that a relatively high total chromosome number, the presence of microchromosomes, the absence of differentiated sex chromosomes and the localization of loci of NORs on microchromosomes can be usually considered plesiomorphic conditions in most families and genera [15,28,38].

Comparative gene mapping also showed that the karyotypes of the squamate common ancestor probably included several microchromosomes and 10 macrochromosome pairs. In turn, the common ancestor of Toxicofora likely had a karyotype composed of six macrochromosome pairs from which originated (via a chromosome fusion and a fission) six and eight macrochromosome pairs in the common ancestor of Iguania and Serpentes, respectively [39,40]. In Iguania, this hypothesis is supported by the high number of different taxa (species, genera and whole families) showing a fixed chromosome formula of 2n = 36 with 6 macro- and 12 microchromosome pairs (Figure 1). Similarly, a karyotype composed of 2n = 36 with 8 macro- and 10 microchromosome pairs is highly conserved among many taxa in Serpentes and it is usually considered the ancestral state of the whole group [41,42,43,44].

## 3. Chromosome Fusions

Fusions are known to be involved in speciation and lineage diversification mainly through the insurgence of errors in meiotic segregation and the reduction in gene flow by the alteration of the recombination pattern [1,45,46]. The evolutionary and phylogenetic impact of chromosome fusions has been described in a wide range of vertebrate and invertebrate taxa (see, e.g., [1,46,47,48].

In squamates, chromosome fusions probably represent the most common category of whole-chromosome rearrangements. In fact, whether in the form of centric fusions or micro- to macrochromosome translocations, they have likely played a major role in the evolution and the diversification of the squamate karyotype at different taxonomic levels [38]. At a high taxonomic level, at least nine chromosome fusions have been hypothesized to have given rise to the ancestral squamate karyotype starting from the chromosome complement of the amniote common ancestor, while five additional fusions led to the formation of the highly conserved six macrochromosome pairs of Iguania [38,39].

At the family level, there are several instances of progressive accumulations of chromosome fusions, eventually leading to a reduction in the total chromosome number, an augment of the number of biarmed (often meta- or submetacentric) elements and a decrease in the number of telocentric elements. Furthermore, chromosome fusions in squamates often involve microchromosomes and may lead toward their progressive disappearance and the transition between discontinuous and continuous karyotypes [19,28].

These processes can be particularly evident in families and genera with species displaying different karyotype structures (e.g., discontinuous and continuous), where these rearrangements can be tracked using different techniques. For example, in Chamaeleonidae, the ancestral karyotype of Iguania (of 2n = 36, with 6 macro- and 12 microchromosome pairs) is ultra-conserved in the genera *Brookesia*, *Palleon* and *Kinyongia* and has also been described in *Calumma* and *Trioceros*, but multiple independent fusions and micro- to macrochromosome translocations produced karyotypes with a lower chromosome number (up to 2n = 20–22, with 1–2 microchromosome pairs) in several evolutionary lineages [19,25]. Similarly, a process of independent reduction in the total chromosome number and progressive microchromosome disappearance by micro- to macrochromosome translocation has been reported in several gecko taxa including the genera *Blaesodactylus*, *Gekko*, *Lygodactylus*, *Matoatoa*, *Paroedura* and *Uroplatus*, (see, e.g., [18,49,50,51,52]). All these clades are characterized by a karyotype composed of 2n = 34–42 mostly telocentric chromosomes and the progressive appearance of biarmed elements by means of chromosome fusions in karyotypes with a relatively lower chromosome number [51,52]. It should be noted that even when leading to a similar karyotype formula these rearrangements can often be non-homologous in different evolutionary lineages, involving distinct micro- and or macrochromosome pairs [19,28].

A similar propensity of small chromosomes to fuse with larger ones has also been recognized in invertebrates. For example, in Lepidoptera, multiple chromosome fusions appear to be linked to an increased background selection and selection against hybrids which in turn have promoted a reduced genetic diversity [53].

In several lizard families and genera, fusions between autosomes and sex chromosomes are also responsible for the formation of multiple-sex-chromosome systems with either male (X_1_X_1_X_2_X_2_/X_1_X_2_Y) or female heterogamety (Z_1_Z_1_Z_2_Z_2_/Z_1_Z_2_W). So far neo-sex-chromosome and multiple-sex-chromosome systems have been described in nine lizard families, but are much rarer in snakes (e.g., in *Bungarus*) [14,15,54]. It has also been hypothesized that some autosomes have a particular predisposition for both turning into neo-sex chromosomes or for fusing with already existing ones. For example, these processes may be favored by a positive selection of translocations of sexual antagonistic loci on sex chromosomes and heterozygosity of the heterogametic sex [55,56]. Female meiotic drive also has a possible impact on fusion between autosomes and sex chromosomes, possibly explaining the unbalanced occurrence of multiple-sex-chromosome systems with male and female heterogamety in squamates [15,57].

Similarly, non-homologous Y/W autosome fusions are also known to have arisen multiple times in closely related evolutionary lineages. For example, Dactyloidae, *Anolis*, *Ctenonotus* and *Norops* share a conserved region of the X chromosome of *Anolis carolinensis*, but fusions involving different autosomes led to the formation of cytogenetically distinct multiple-sex-chromosome systems in the latter two genera [58].

## 4. Chromosome Fissions

Similarly to chromosome fusions, due to fissions, chromosomal heterozygotes may suffer from meiotic errors or sterility (or reduced fertility) and/or be heavily selected against (see, e.g., [59,60]). When multiple chromosome fissions occur they lead to a dramatic increase in the chromosome number which can be erroneously interpreted as a whole genome duplication [61].

Available karyological data suggest that chromosome fissions are also widespread in the squamate phylogeny, but they are probably rarer than fusions. Nevertheless, they are responsible for some of the most striking examples of chromosome diversification in different taxonomic groups. In squamates, multiple chromosome fissions have been described in many lizard families including Anguidae, Scincidae, Iguanidae, Gekkonidae and Phrynosomatidae, where they often led to the formation of karyotypes of 2n > 36 with several acrocentric elements (see, e.g., [62,63]).

The highest diploid chromosome number found in lizards (of 2n = 62, with mostly acrocentric elements) originated two times independently, in *R. brevicaudatus* (Chamaeleonidae) and *N. ablephara* (Gymnophtalmidae), probably following a similar pathway of multiple Robertsonian centric fissions starting from karyotypes with a lower total chromosome number and several biarmed elements [24,25,64]. A similar evolutionary pathway has been reported for various representatives of the subfamily Teiinae, which are characterized by a chromosome formula of 2n = 46–56 with several acrocentric chromosomes [22,65].

Interestingly, in Lacertidae, 10 macrochromosome pairs of *Zootoca vivipara* and *Podarcis muralis* align to the 5, highly conserved, largest chromosome pairs of *Salvator merianae* (Teiidae), *Intellagama lesueurii* (Agamidae) and snakes, suggesting the occurrence of several centric fissions [28].

In Serpentes, the hypothesized ancestral snake karyotype (of 2n = 36, with 8 macro- and 10 microchromosome pairs) is highly conserved in several clades, but several chromosome fissions have probably occurred in the evolution of the highly divergent karyotype of several families of Elapoidea (e.g., Elapidae, Psammophiidae, Lamprophiidae and Pseudoxyrhophiidae) which is composed of 2n = 42–48 and many acrocentric elements [41,66].

Similarly, in Boidae, the putative snake ancestral karyotype of 2n = 36 is conserved in the genera *Boa*, *Eunectes*, *Chilabothrus* and *Epicrates*, but a higher chromosome number (2n = 40–44) has been described in *Corallus* [44,67]. These divergent karyotype formulas probably resulted from several chromosome-centric fissions which decreased the number of meta- and submetacentric elements and increased the count of acrocentric chromosomes. Notably, the primitive undifferentiated sex chromosomes (XY) originally found in *Boa* appear to be conserved in all the species with 2n = 36 chromosomes, while in *Corallus caninus* (with 2n = 44) they were split by a chromosome fission into the small acrocentric chromosome pairs, 11 and 12 [44]. There is currently no evidence on whether a single or a multiple-sex-chromosome system occurs in the genus *Corallus*.

Recent studies suggest that chromosome fissions also had an important role in the sex chromosome evolution of amniotes. In fact, it has been hypothesized that the sex chromosomes of different evolutionary lineages originated from multiple fission events from an ancestral amniote super-sex chromosome [68,69]. This view is supported by phylogenetically unrelated sex chromosomes sharing partial linkage homologies among various taxonomic groups. However, sex chromosomes are largely non-homologous in different vertebrate evolutionary lineages [70], and particular regions showing partial homology among different taxonomic groups might have been co-opted multiple times independently for a sex-determining or sex-linked function [68].

## 5. Chromosome Inversions

Chromosome inversions have been traditionally recognized as sources of rapid and dramatic genetic variability and are widely associated with divergence between populations and ecotypes, homoploid hybrid speciation (HHP) (speciation without change in the chromosome number) and phylogenetic lineage diversification in various animal taxa (see, e.g., [71,72]).

The multiple evolutionary roles of chromosome inversions include selection against and reduced fertility of heterokaryotypes, suppression of recombination, protection of adaptive alleles from recombination and differential accumulation of divergently selected loci leading to genetic diversification of locally adapted populations [73,74].

However, inversions are often more difficult to cytogenetically detect and localize than interchromosomal changes which result in a variation in the total chromosome number and several insights on their evolutionary roles in animals come from *Drosophila*, *Anopheles* and other Diptera (e.g., [75,76,77]). Nevertheless, the occurrence of autosomal inversions and their possible taxonomic and evolutionary consequences have been described in several squamate families including Boidae, Dactyloidae, Gekkonidae, Gymnophthalmidae, Hydrophiinae, Phrynosomatidae, Phyllodactylidae and Varanidae [21,67,78,79,80,81,82].

Molecular analyses of the genus *Sceloporus* evidenced a correlation between chromosome changes and rapid phylogenetic divergence [2]. The plateau fence lizard *Sceloporus undulatus* species complex (Phrynosomatidae), shows a karyotype composed of 2n = 22 chromosomes and a polymorphic inversion on chromosome pair 7. Different configurations of pair 7 characterize different populations with distinct geographical distributions and individuals in hybrid zones show heteromorphic inversions at pair 7 [83]. It has been hypothesized that the alternative configurations of pair 7 might have a role in the ecological and evolutionary diversification of several genetic lineages, but the underlying mechanisms remain unclear.

A recent cytogenetic comparison between *Hemidactylus mercatorius* and *Hemidactylus mabouia* (Gekkonidae), which were previously considered synonyms, revealed the same chromosome number (2n = 42), but a different morphology of several chromosome pairs, which likely originated from multiple inversions [82]. The relatively high number of inversions putatively involved in the chromosome diversification of the two species (n = 5) appears of particular interest considering their sister-species status and suggests a relevant role of these rearrangements in the delimitation of independent evolutionary lineages.

In Phyllodactylidae, the genus *Phyllopezus* comprises karyotypes of 2n = 38–40 which are mostly distinguishable in different species and subspecies by the different number of telocentric and metacentric elements, which are probably derived from a combination of centric fusions and pericentric inversions [84].

Chromosomal inversions might be involved in the diversification and loss of recombination of sex chromosomes, often coupled with heterochromatinization and driven by sexually antagonistic selection (sexual specialization). In fact, one of the hypothesized pathways of the formation of heteromorphic sex chromosomes involves the insurgence of a favorable sex-linked inversion in the proto-Y/W chromosome. A novel allele might produce a fitness increase in either males or females and a decrease in fitness in the other sex, becoming favorably selected for linkage with the sex-determining locus on the heterogametic sex chromosome [85]. Alternatively, inversions may avoid homozygous expression of deleterious mutations in partial linkage with sex-determining loci [86].

In Hydrophiinae, a large inversion (of about 35 Mb) was localized on the Z chromosome of *Hydrophis cyanocinctus* and *H. curtus*, suggesting the occurrence of an ongoing evolutionary pathway of heteromorphic sex chromosome diversification (ZW) by suppressed recombination [79]. This rearrangement involves more than 43% of the Z chromosome length (and several Z-linked loci), potentially influencing divergent phenotypic adaptations between the two species [79].

## 6. Evolutionary Perspectives

Four different general karyotype evolutionary trends and pathways can be generalized in squamates and, more in general, in vertebrates: karyotype evolutionary stasis; decrease in the total chromosome number (by chromosome fusions); increase in the total chromosome number (by chromosome fissions) and lineage diversification by homoploid speciation (see, e.g., [59,87,88,89]).

Karyotype stasis has been defined as the invariability in ploidy, chromosome number, general morphology and genome organization during phylogenetic diversification [59]. Comparable examples of karyological evolutionary stability have been reported in plants and several clades of the main vertebrate evolutionary lineages, including fish, birds and amphibians (see, e.g., [59,90,91]).

It should be noted that chromosome number and morphology cannot be used alone as a proof of synteny because karyotypes showing a very similar structure may include cryptic rearrangements (e.g., paracentric inversions). However, available data suggest that morphologically highly conserved karyotypes retain similar linkage groups and general genome structure [59,92].

For example, in the family Anguidae, five *Anguis* species, as well as *Pseudopus apodus,* have a conserved karyotype of 2n = 44 (with 10 macro- and 14 microchromosome pairs and no differentiated sex chromosomes). Among these species, subtle variations in chromosome morphology are due to a differential genomic distribution of DNA repeats, but chromosome homology is highly conserved between *Anguis* and *Pseudopus* [17,93].

Experimental analyses on relatively simple model organisms such as yeast have demonstrated that when chromosome changes do occur, and are not heavily selected against, they are potentially able to produce reproductive isolation and speciation [60,94]. These effects can be more profound in larger genomes with several chromosome pairs, where multiple chromosome rearrangements may produce an extensive genome reshuffling [95]. Furthermore, distinct kinds of inter- (e.g., fusions and/or fissions) and intrachromosomal rearrangements (e.g., inversions) may co-accumulate in a karyotype or a chromosome pair and contribute to the establishment of a reproductive barrier (e.g., [96]).

There are several possible explanations for how novel chromosome rearrangements became fixed in natural populations. The simplest scenario involves fixation via genetic drift and/or inbreeding in small-sized populations [1]. Alternatively, particular chromosome rearrangements could be preferentially transmitted if linked to meiotic drive and occur in future generations with an increased frequency [97]. This hypothesis is supported by the frequent localization of meiotic-drive-linked loci within inverted chromosome regions [98]. Other research suggests, instead, a predominant adaptive role of chromosome rearrangements, which therefore would become fixed by natural selection [99]. For example, the chromosomal polymorphism and karyotype variability in *Anolis* and *Norops* may be associated with adaptive radiation and speciation [100]. These different hypotheses are not mutually exclusive and multiple mechanisms can possibly influence different rearrangements in the same evolutionary lineage [5].

Regardless of the mechanisms involved in the promotion and fixation of chromosome changes, experimental evidence highlights that certain clades are more prone to a particular kind of rearrangement. As discussed above, a decrease in the chromosome number via chromosome fusions and micro- to macrochromosome translocations is overall the more frequently observed karyotype dynamic in squamates, but the opposite pathway (increase in chromosome number by fissions) nevertheless influences the chromosome diversification of several lineages. Moreover, different evolutionary trends may coexist in different clades of the same taxonomic group.

**Figure 3:**
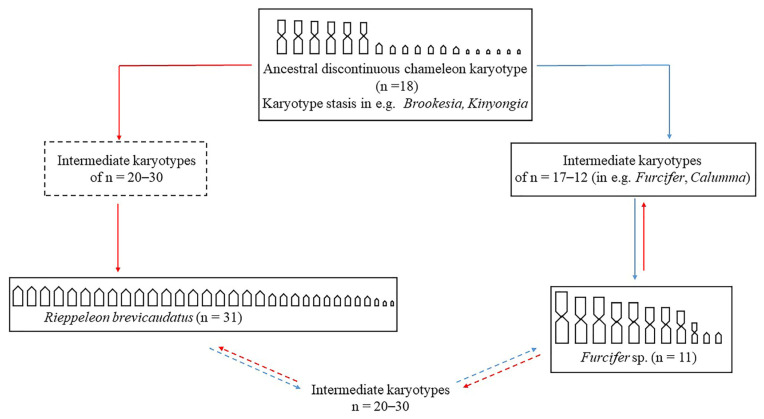
Different evolutionary trends and hypothesized circular karyotype evolutionary pathways in chameleons. Solid boxes = observed karyotype states. Dashed boxes = putative karyotype states. Solid lines = documented chromosome rearrangements. Dashed lines = hypothesized chromosome rearrangements. Blue lines = chromosome fusions. Red lines = chromosome fissions.

Recent macroevolutionary analysis of the chromosome diversification in the family Chamaeleonidae showed the occurrence of at least three different evolutionary dynamics, including: (i) karyotype evolutionary stasis (of the hypothesized ancestral 2n = 36 with 6 macro- and 12 microchromosome pairs) in *Brookesia*, *Palleon* and *Kinyongia*; (ii) generalized decrease in the chromosome number in most other genera (up to 2n = 20–24) and (iii) increase in the chromosome number by chromosome fissions in a limited number of species (*R*. *brevicaudatus*, *Calumma amber* and *C. tarzan*) [19] (Figure 3).

Interestingly, a relatively low chromosome number was achieved independently in several chameleon genera, suggesting the occurrence of a convergent karyotype evolution, which is consistent with the karyotypic orthoselection model proposed by White [101]. According to White’s model, the accumulation of similar chromosome rearrangements in any given lineage is not random but is explained either by environmental selection or intrinsic chromosomal properties and would eventually lead to convergent karyotype structures [101]. In the case of the family Chamaeleonidae, there was no correlation between natural history traits and karyotypes, possibly indicating that intrinsic genomic properties (or selection of a favorable genome structure) led to the independent acquisition of a convergent karyotype organization [19].

The occurrence of different chromosome evolutionary trends in chameleons highlights the evolutionary plasticity of squamate karyotypes and may also suggest the potential for their general morphology and genome organization to evolve circularly. In fact, morphologically extreme karyotypes, for example, of various *Furcifer* species (of 2n = 22–24, all biarmed macro- and 1–2 microchromosome pairs) and *R. brevicaudatus* (of 2n = 62, all acrocentric chromosomes), should not be interpreted as evolutionary dead ends, but as steps in a virtual “circular karyotype evolution” (Figure 3). A limit to this hypothesis comes from the observation that the evolutionary trajectory of microchromosomes usually appears unidirectional, ending with their fusion with other micro- or macrochromosome pairs [28]. However, the formation of neo-microchromosome pairs via chromosome fission is not unknown in squamates [28], thus allowing transitions between discontinuous and continuous karyotypes and vice versa.

Other examples of putative karyotype circular evolution can be made at different taxonomic levels. In Gekkota, tendencies of karyotype stasis, decrease and increase in the chromosome number, as well as the occurrence of karyotypes mostly composed of either acrocentric or biarmed elements, have been described in different clades (e.g., [18,49]). This evidence suggests the possibility of a continuous genome reshuffling and reoccurring morphological karyotype structures over evolutionary times.

New insights into chromosomes and, more in general, into biological evolution, come from recent theories advocating for “system inheritance” models beyond the classical assumptions of gene-centric evolutionary mechanisms [102]. In fact, the link between the genetic code and gene-driven macroevolution presents several limitations and the informational relationship between small microevolutionary novelties at the sequence level and large macroevolutionary changes still requires more exhaustive explanations (see, e.g., [103]). The “karyotype coding” hypothesis (see [102,104,105]) proposes that the whole genome structural organization (e.g., morphological and topological structure including gene order) represents a higher code, whose changes are able to drive macroevolutionary dynamics. In the context of the karyotype coding hypothesis, the circular karyotype evolution represents a potential mechanism for a continuous reshuffling of the code.

## 7. Conclusions

Squamate reptiles are karyologically very diverse and represent exceptional model organisms in evolutionary cytogenetics. Chromosome diversity in squamates involves several variable features, including general karyotype structure (continuous or discontinuous) and chromosome number and morphology, which are non-randomly distributed across their phylogeny. The main categories of balanced chromosome rearrangements have all been documented as evolutionary drivers in different evolutionary lineages and taxonomic levels. Furthermore, distinct karyotype evolutionary trends and diversification pathways are known to occur with different frequencies in different evolutionary lineages, also coexisting in some groups. Overall, a decrease in the chromosome number by chromosome fusions (and microchromosome translocation) seems the most common dynamic in the karyotype evolution of squamates, but karyotype stasis and increase in the chromosome number are also known to occur in several evolutionary lineages at different taxonomic ranks. The complex patterns emerging from the chromosome diversity of squamates are particularly suited for testing traditional and novel hypotheses on karyotype evolution.

## 8. Future Directions

Despite the high chromosome diversity of squamate reptiles, the available karyotype data are still limited when compared with their taxonomic diversity. In fact, the relatively low number of described karyotypes remains the major limit for a deeper understanding of the chromosomal evolution of squamates. Furthermore, a high proportion of available chromosome data on squamates comes only from standard karyotyping. At a species level, further studies employing chromosomic approaches combined with traditional staining, banding techniques and molecular cytogenetic are still needed to better describe and assess the diversity of squamate karyotypes. From a macroevolutionary point of view, interdisciplinary approaches linking cytogenetics, molecular phylogenetics and phylogenetic comparative methods would be particularly appropriate for hypothesis testing of suggested processes of karyotype evolution at high taxonomic levels.

## Data Availability

No new data were created or analyzed in this study. Data sharing is not applicable to this article.
